# Study on the Thermoregulation Mechanism of Temperature Insensitive Asphalt Pavement

**DOI:** 10.3390/ma18184326

**Published:** 2025-09-16

**Authors:** Yongjun Yang, Xiaojun Cheng, Yang Qi, Meng Guo, Shanglin Song, Xiaoming Kou, Fukui Zhang

**Affiliations:** 1 Gansu Gannan New Century Road and Bridge Co., Ltd., Gannan 747000, China; yangyj9418@163.com; 2 Scientific Observation and Research Base of Transport Industry of Long Term Performance of Highway Infrastructure in Northwest Cold and Arid Regions, Dunhuang 736200, China; 3 State Key Laboratory of Bridge Engineering Safety and Resilience, Beijing University of Technology, Beijing 100124, China; chengxj@emails.bjut.edu.cn (X.C.); qiy@emails.bjut.edu.cn (Y.Q.); 4 The Key Laboratory of Urban Security and Disaster Engineering of Ministry of Education, Beijing University of Technology, Beijing 100124, China; 5 Gansu Provincial Highway Development Group Co., Ltd., Lanzhou 730070, China; 18193106299@163.com (S.S.); 17801052381@163.com (X.K.); 13993109118@163.com (F.Z.)

**Keywords:** composite phase change material, asphalt mixtures, temperature regulation mechanism, road performance

## Abstract

Incorporating phase change materials into asphalt concrete and utilizing phase change heat transfer to control the temperature of asphalt pavement can effectively reduce the impact of high temperatures on the durability of asphalt pavement. In this study, microencapsulated composite phase change materials were prepared using calcium alginate and polyethylene glycol (PEG) 1500 and mixed into SMA-13 Marshall specimens for indoor high-temperature tests. The test results show that the temperature of the specimen was reduced by about 1.5 °C when the doping amount of the composite phase change material was 2.4% and the oven temperature was 60 °C. In order to further investigate the application of phase change energy storage materials in asphalt pavement structure, this study used Comsol finite element software to simulate the summer temperature field of the asphalt surface layer. A three-layer asphalt pavement model consisting of 4 cm SMA-13, 6 cm AC-20, and 8 cm AC-25 was established to study the effect of phase change materials on the temperature change in the pavement. The results of this study show that adding 2.4% of the composite phase change material to each of the top and middle surface layers kept the temperature of all pavement layers outside of the temperature range in which the asphalt’s dynamic stability plunges.

## 1. Introduction

Asphalt pavement is used extensively throughout the world due to its advantages of driving comfort, short recuperation time after construction, fast opening to traffic, and convenient renovation. In China, nearly 70% of roads use asphalt as the surface layer, and the utilization rate in high-grade pavement is more than 95%. As an artificial paving material, asphalt pavement is subjected to climatic changes and vehicle loads, which can lead to the deterioration and decay of asphalt materials. Since asphalt is a temperature-sensitive material that exhibits viscous-elastic-plastic properties with changes in temperature, changes in temperature can negatively affect asphalt materials during actual use [1,2,3]. In a high-temperature environment, the temperature will have a huge impact on asphalt pavement, mainly as follows: accelerating asphalt aging and inducing pavement deformation, rutting, congestion, and other diseases. It is generally believed that asphalt pavement rutting is caused by the lack of shear strength of asphalt mixtures under high-temperature conditions, as shown by studies [4,5] when the temperature is higher than 55 °C. Asphalt mixture rutting develops at a centimeter rate; therefore, an effective way to control the generation and development of pavement rutting disease is to reduce the pavement temperature, thereby reducing the external environment of the pavement to produce disease; in a low-temperature environment, low temperature will also have an adverse effect on the pavement, mainly by inducing asphalt pavement cracking, reducing the service life of the pavement. In rainy and snowy weather, too low a pavement temperature will slow the elimination of the rain and snow, which will lead to slippery road surfaces, resulting in traffic accidents, for example, due to loss of vehicle control. The regulation of asphalt pavement temperature, such that the pavement temperature does not become too low at low temperatures, is an effective manner of reducing cracking and ice condensation. In order to reduce the impact of temperature on asphalt materials and the decay rate of the asphalt mixture under the action of temperature, in recent years, scholars at home and abroad have proposed a number of improvement measures based on the characteristics of the asphalt, mainly from two aspects. On the one hand, they have proposed improving the pavement material’s resistance to high temperatures and the low-temperature performance and reducing the temperature sensitivity of the material. To these ends, scholars have focused on the research and development of rutting-resistant asphalt mixture, cracking-resistant asphalt mixture, and so on to address the temperature change passively. However, although a certain effect has been achieved, it is not ideal. On the other hand, research has aimed to improve the temperature field of asphalt pavement, thereby improving the working temperature range of asphalt mixtures. Currently available technologies mainly include heat reflection, pavement water retention and cooling, thermal resistance pavement, and pavement energy conversion [6,7,8,9,10,11].

With the continuous development of technology, thermoregulation with phase change materials has become a research direction for scholars at home and abroad. Phase change materials, also known as phase change energy storage materials, are latent heat energy storage materials. They are a type of energy storage material that absorbs or releases heat through their own phase state transformation. Introducing phase change materials into asphalt pavement can slow the heating and cooling rates of asphalt by absorbing and releasing heat through the phase transformation of the phase change materials themselves. At present, phase change materials (PCMs) are widely utilized in aerospace, refrigeration, and other fields [12,13,14,15,16]. In construction, phase change energy storage was used to treat building materials (such as gypsum board, wallboard, concrete components, etc.) in the 1990s, and the research and application of PCMs in concrete test blocks, gypsum wallboard, and other building materials was very active. In 1999, a new type of building material, solid–liquid eutectic phase change material (PCM), was successfully developed abroad. This PCM can be poured into wall panels or lightweight precast concrete panels to maintain a suitable indoor temperature [17,18,19,20]. In addition, many companies in Europe and the United States use PCMs to produce and sell outdoor communication wiring equipment and power transformer equipment in a special cabin, which can be maintained at a suitable working temperature in both winter and summer. Tseng et al. used paraffin, n-pentadecane, and n-octadecane as phase change materials and urea–formaldehyde resin as a wrapping wall material. Following the preparation of a microcapsule composite phase change material, their study showed that the microcapsule on the solid–liquid phase change material core samples had good wrapping, and the latent heat of the phase change was approximately 109 J/g [21]. Cho et al. used n-octadecane as the phase change material and prepared microcapsule phase change materials with polyurea walls using the interfacial polymerization method. The results of their DSC experiments showed that the phase change temperature range of the microcapsule composite phase change materials was 29–30 °C, and the latent heat of the phase change was up to 110 J/g [22]. Zhang et al. created EG/PEG composite phase change material by adsorbing PEG into the pores in EG using the vacuum impregnation method, and the composite was found to have a good encapsulation effect through SEM and Fourier Transform Infrared Spectroscopy (FTIR) experimental studies when the mass ratio of EG and PEG in the composite phase change material was not less than 1:7 [23]. Muhammad Rafiq Kakar et al. [24] prepared tetradecane phase change microcapsules and added them to asphalt to obtain low-temperature phase change modified asphalt with a phase change temperature of 5 °C and an enthalpy of 35 J/g. Kun et al. [25] synthesized low-temperature polyurethane solid–solid phase change materials with phase change temperatures ranging from −5 °C to −12.5 °C and an enthalpy of 47 J/g using the prepolymer method, and the cooling rate of the polyurethane solid–solid phase change-modified asphalt was significantly reduced compared to the asphalt without the phase change material. Deng et al. proposed a systematic numerical simulation framework to quantify the effect of phase change materials (PCMs) on the early rutting performance of asphalt concrete pavements. The pavement system was characterized and modeled in terms of material, structure, environment, and traffic loading. The results showed that the cumulative rutting depth in asphalt concrete with only 3% PEG/SiO_2_ added was reduced by 4% after the first week of pavement use [24,25,26]. Gao et al. prepared silica composite phase change materials using the sol-gel method, developed a finite element heat transfer model by measuring the boundary conditions, and predicted and analyzed its conditioning effect [27]. Amini et al. [28] showed that nano-CuO as a modified asphalt binder can store heat via heat absorption/excretion to prevent sudden temperature increase/decrease, and using nano-CuO as a PCM improved the Tmg of the asphalt binder; Tpc decreased by a few degrees and the C-value decreased by more than 7 °C compared to those values of pure asphalt binder. Cheng et al. [29] showed that with the increase in polyethylene glycol polyacrylamide graft copolymer (PPGC) phase change material dosing, the immersion Marshall test and residual stability of asphalt mixtures showed an increasing and then decreasing trend—the smaller the aggregate particle size of the asphalt mixture, the earlier the maximum value of immersion Marshall appeared. This indicates that a smaller particle size leads to a denser mixture and better energy transfer in the mixture, relatively.

Currently, there is an increasing focus on the research and development of dual-phase change thermoregulation materials for asphalt pavement [30,31,32]. However, current studies on the application of phase change materials in asphalt mixtures mainly explore the thermoregulation effect of composite phase change materials with phase change temperatures on asphalt mixtures through experimental research, which has certain limitations. Conversely, simulation research can produce the pavement temperature field under different scenarios and assumptions. 

Based on the above, the objectives of this research concern determining the asphalt mixture material for the study by selecting the composite shape of the phase change material suitable for pavement material and the asphalt mixture ratio design, as well as simulating the temperature regulation effect of the phase change material by measuring the internal temperature change in asphalt mixture while warming up indoors. Thus, this study carried out finite element simulation analysis on the asphalt mixture and asphalt pavement structure internal temperature field analysis and compared the measured data. The results of this study are significant as references for the development of phase change materials for pavement and the research and application of asphalt pavement temperature regulation technology.

## 2. Experimental Materials and Methods

### 2.1. Experimental Raw Materials

#### 2.1.1. Selection and Preparation of Phase Change Materials

In view of the application objectives, application scenarios, and functional requirements of phase change materials, phase change materials applied to asphalt pavements should meet the following requirements:

1. Phase transition temperature

This is the first factor considered in the selection of phase change materials. Suitable phase change materials should be selected based on the specific working environment of the pavement, mainly considering the temperature conditions; the material’s phase change working temperature should help achieve the proposed temperature regulation of the pavement.

2. Good stability of phase change cycle

Phase change heat storage materials in asphalt pavement should be robust to repeated traffic loads and natural environmental factors. Such materials can give full play to the phase change heat storage function to realize the reversible phase change cycle, generally through appropriate encapsulation materials and a suitable encapsulation process.

3. Good thermal stability (no decomposition at 200 °C)

To meet the requirements of the high-temperature mixing and construction of asphalt mixtures, hot-mix hot-paving asphalt mixtures are usually heated to 150~170 °C, with the mineral aggregate heated to 180~190 °C. Therefore, the thermal stability of the materials used must withstand the high-temperature process of hot mixing and hot paving. Through this high-temperature mixing, paving, and rolling process, thermal decomposition and other chemical reactions do not occur, which is also ensured through the selection of encapsulated and phase change materials.

4. It has good chemical compatibility with asphalt

Phase change materials and asphalt mixtures have good chemical compatibility, such that under the high-temperature conditions of the mixing and paving, the materials do not deteriorate and they maintain their original nature.

Based on the above factors, including the working temperature range of the asphalt pavement and asphalt pavement material production, the paving process and its requirements, comprehensive phase change materials, and asphalt compatibility, a molecular weight of 1500 polyethylene glycol (PEG1500) was chosen for the core material. All these materials (PEG1500) are produced by Jiangsu Haian Petrochemical Plant. In order to shape the material and ensure phase change stability and thermal stability, calcium alginate, a reaction product of sodium alginate and calcium chloride, was used as the encapsulation material.

The above core and encapsulation materials were prepared as a composite shaped phase change material via ion substitution. This composite phase change material comprised polyethylene glycol (PEG1500) and calcium alginate [33]. In this material, PEG1500 is used as a phase change core material, mainly to regulate the temperature. In contrast, calcium alginate, a reaction product of sodium alginate and calcium chloride, acts as a wall material to encapsulate PEG1500, thus ensuring that the liquid phase does not leak during the phase change process. It also ensures the phase change material has certain stability and durability during preparation and use. The specific preparation process of the shaped phase change material follows:

(1) Sodium alginate powder and calcium chloride were added to deionized water at a temperature of 40 °C and stirred continuously with a glass rod during the addition process until the sodium alginate powder and calcium chloride particles were fully dissolved to form a 2.5 wt% sodium alginate solution and a 2.5 wt% calcium chloride solution.

(2) Liquid PEG1500 was added to the sodium alginate solution at a temperature of 80 °C. The temperature was maintained and stirred at 1000 rpm until homogeneous mixing was achieved to obtain a blend of both components.

(3) Calcium chloride solution was dropped into the co-mixture of calcium alginate and PEG1500 at a certain rate. It was left to stand for 8 h to obtain a wet composite phase change material.

(4) The wet composite capsule was cleaned and dried to obtain the dry composite phase change material.

**Figure 1:**
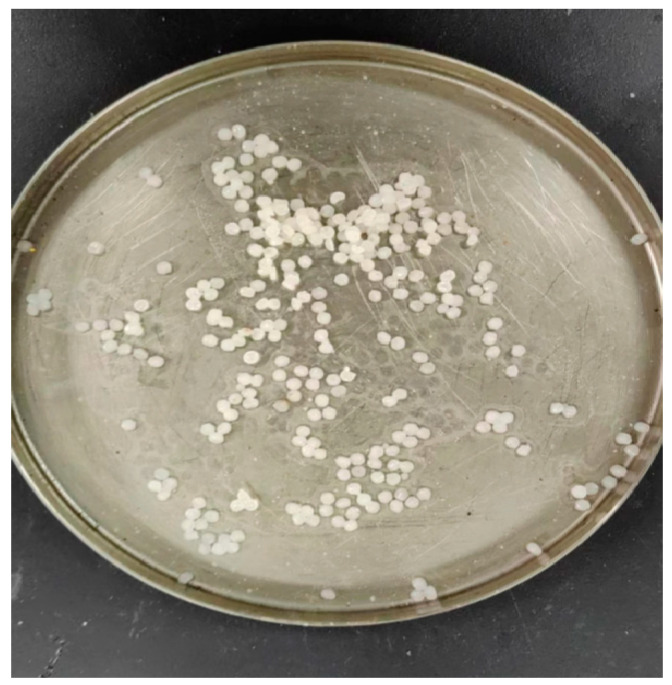
Composite shaped phase change material.

The prepared composite shaped phase change material was a white granular solid, as shown in Figure 1, with a hard texture. It can be used as an asphalt additive, and its apparent density is 1.08–1.21 g/cm^3^.

#### 2.1.2. Mineral Gradation

**Figure 2:**
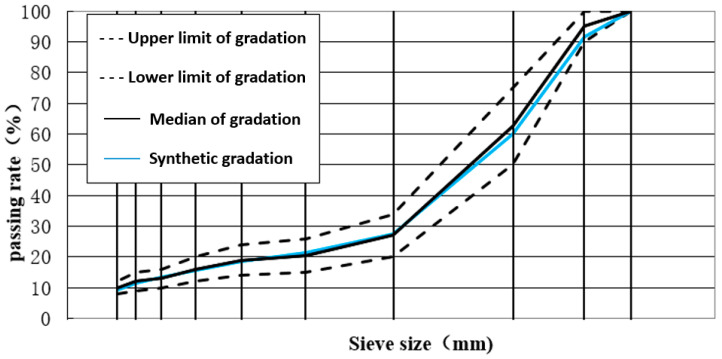
Mineral and synthetic grading plan.

A test was conducted on the asphalt mixture type for the surface layer of asphalt pavement using the commonly used SMA-13 mixture (the materials are from Beijing Municipal Road & Bridge Co., Ltd., Beijing, China). The Marshall method was used to evaluate the asphalt mixture ratio according to the SMA-13 range of mineral gradation requirements; Table 1 lists the mineral gradation and synthetic gradation scales, and Figure 2 shows the synthetic gradation.

#### 2.1.3. The Optimal Asphalt Content of SBS-Modified Asphalt Was Selected

The asphalt was tested according to the standards of China’s “Highway Asphalt Pavement Construction Technical Specification” (JTG F40-2004) requirements, and “Highway Engineering Asphalt and Asphalt Mixture Testing Procedures” (JTG E20-2011) [34,35], and the results are shown in Table 2.

The volumetric method was used to assess the asphalt mixture ratio design, with double-sided compaction conducted 50 times to prepare the Marshall specimens. The VMA, VCAmix, and other indicators were measured, based on the chosen asphalt mixture ratio; the optimal oil–rock ratio of SMA-13 was determined to be 6.0%, and the results of the ratio design are shown in Table 3.

Data regarding the design of the SMA Marshall test ratios and the corresponding technical requirements are shown in Table 4.

According to the requirements, the road performance results of the SMA-13 mixtures at the optimal oil–gravel ratio were examined and are shown in Table 5.

As can be seen from Table 5, its indicators are in line with the “highway asphalt pavement construction technical specifications” (JTG F40-2004) [34]. The performance of the designed SMA-13 mixture meets the road technical requirements, meaning it can be used in the road. Follow-up research should use the asphalt mixture to add phase change materials in temperature-related experimental studies.

### 2.2. Experimental Methods

#### 2.2.1. Preparation of Phase Change Asphalt Mixture

In the asphalt mixture ratio design, the composition of the mixture was determined based on the preparation of the phase change asphalt mixture. The shape of the phase change material, as an external dopant, was developed according to a certain asphalt mass ratio and mineral powder mixed directly into the asphalt mixture. In order to analyze the influence of the amount of phase change material on the cooling effect, based on previous research experience [36], the following ratios of admixture to asphalt were used: 10%, 20%, 30%, and 40%. Since the asphalt dosage of the SMA-13 asphalt mixture in this study was 6.0%, when the phase change material in the asphalt accounted for 10%, 20%, 30%, and 40% of the mass ratio, its proportion of asphalt mixture in the dosage was 0.6%, 1.2%, 1.8%, and 2.4%, respectively. The process of phase change asphalt mixture preparation is as follows:

(1) Firstly, three kinds of mineral materials, with particle sizes of 10–15 mm, 5–10 mm and 0–3 mm, were put into a drying oven at 180 °C and heated for more than 4 h to dry the moisture completely. The asphalt was heated in the oven at 165 ± 5 °C until flow state.

(2) According to the proportion determined by the ratio design of the three minerals and fibers added to the mixing pot, dry mixing was performed for 90 s. Then, the flowing asphalt was added and mixed for 90 s. Finally, the mineral powder and the shaped phase change material were mixed together via pot mixing for 90 s.

(3) Marshall specimens were prepared using the percussion method and compacted 50 times on both sides of a standard compactor.

(4) The specimens were maintained at room temperature for 2 days and then demolded.

#### 2.2.2. Experimental Method of Indoor Temperature Adjustment Effect of Phase Change Asphalt Mixture

**Figure 3:**
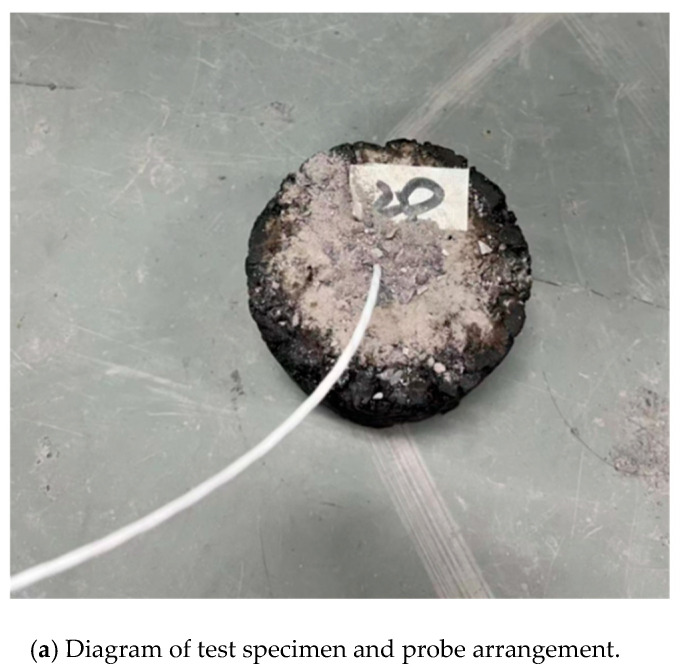

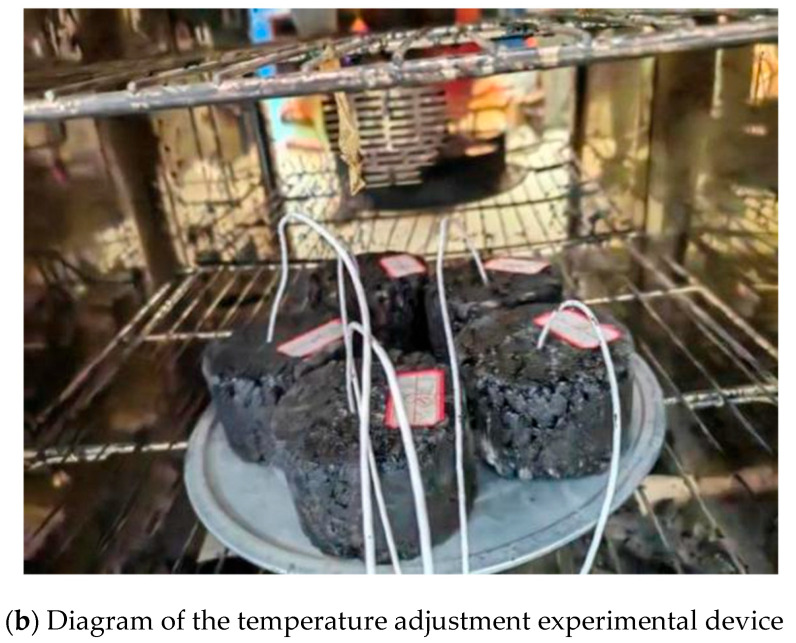
Test specimens and devices.

A standard Marshall specimen of asphalt mixture was molded using the compaction method, with a diameter of 101.6 mm and a height of 63.5 mm. In order to accurately measure the temperature change in the asphalt mixture, a temperature sensor was used to test the temperature. The temperature detection point was set at the center of the specimen. The test method is shown in Figure 3.

(1) In the upper surface of the specimen at the center of the vertical drilling circle, the depth was about 4.5 cm and the length was 2.5 cm. A temperature sensor probe was placed into the drilled holes, backfilling the compaction with mineral powder; an installation schematic is shown in Figure 3a.

(2) The mixture specimen was buried with the temperature sensor in an oven, which was used to warm it up. The temperature recorder connected to the temperature sensor recorded the temperature change inside each mixture specimen. The test specimen and test setup are shown in Figure 3b.

(3) After the temperature of the specimen stabilized, it was removed from the oven and placed in the room to cool down, and the temperature was recorded.

#### 2.2.3. Simulation Method of Indoor Temperature Regulation Effect of Phase Change Asphalt Mixtures

This study used the Comsol Multiphisics 6.0 software heat transfer module for transient studies. First of all, the standard Marshall specimen size was used to establish a geometric model, with a radius of 50.8 mm, a 63.5 mm high cylinder, and coordinates (0, 0, 0). Then, the material parameters were imported, and the basic material properties are shown in Table 6. The density was taken from the density of the Marshall specimen measured in the actual test (Table 6).

The phase change material module was added to this model.

In practice, PCM is uniformly mixed in the asphalt mixture and can be regarded as part of the mineral powder. However, Comsol software, when adding PCM, considers the whole specimen to be completely composed of PCM by default, which is obviously inconsistent with the actual situation; therefore, the latent heat of the PCM was converted in this study. The conversion formula is shown in (1):

(1) $${L^{\prime }}_{1\to 2}=\frac{L_{1\to 2}\cdot x}{1000}$$

where ${L^{\prime }}_{1\to 2}$ is the converted latent heat of the phase change of the PCM from phase 1 to phase 2 (KJ/kg); $L_{1\to 2}$ is the latent heat of phase transition from phase 1 to phase 2 per kilogram of PCM (KJ/kg); and x is the mass of the PCM added in the experiment (g). In this study, phases 1 and 2 are the solid and liquid phases, respectively. For consistency with the actual experiments, the same proportions of 10%, 20%, 30%, and 40% of asphalt mass, i.e., 0.6%, 1.2%, 1.8%, and 2.4% of asphalt concrete, were used in Comsol. The latent heat of the phase change for different PCM proportions is shown in Table 7 below.

#### 2.2.4. Establishment of Outdoor Temperature Field Model of Phase Change Asphalt Pavement

In order to more realistically simulate the working performance of PCM in practice [37], this study chose to establish a three-layer asphalt pavement structure as the object of study, and its pavement structure consisted of the following: 4 cm SMA-13 as the upper layer, 6 cm AC-20 medium-grained asphalt concrete as the middle surface layer, and 8 cm AC-25 coarse-grained asphalt concrete as the lower layer. The performance of each layer of the material is shown in Table 8.

After determining the material parameters of each layer, a model with dimensions of 30 cm × 30 cm × 18 cm was created in Comsol. For the convenience of carrying out the study, the simulations were carried out assuming that each asphalt surface layer material was homogeneous and isotropic. Physical parameters such as density, thermal conductivity, and the constant pressure heat capacity of the material do not change with the change in temperature. In addition, according to the meteorological data, the heat flux of the sun to the ground was set to be 800 w/m^2^. At the same time, the ambient radiant temperature was set to be 35 °C, and the road test specimens were insulated at the bottom, and the road-side test specimens were insulated during the setup so that it could the most unfavorable situation of the test specimens under sunny and hot weather in summer simulate as closely as possible.

**Figure 4:**
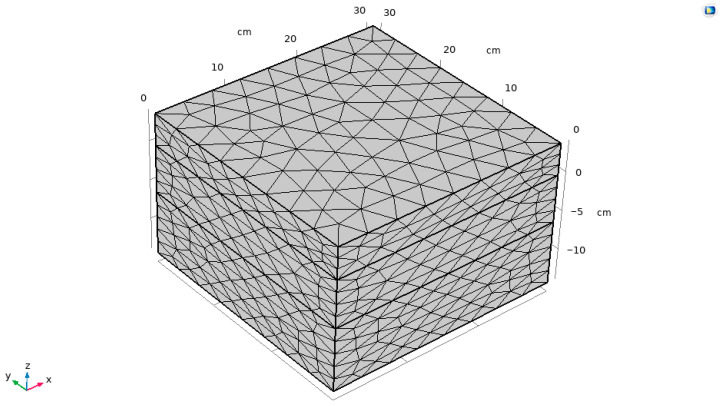
Finite element geometry model and meshing.

The objective of this study is to evaluate the variation in asphalt pavement with external temperature in three layers. Because asphalt pavement transfers heat downward from the pavement during heat transfer, it is necessary to control the mesh size manually along the thickness direction to a smaller value when meshing to improve the accuracy of calculating the temperature at different thicknesses. With a finer mesh, the calculation of the temperature field can be improved to verify the accuracy. For the finite element calculations of the specimen, triangular cells were used for discretization. The maximum cell side length was about 0.01 m, and a finite element schematic is shown in Figure 4.

## 3. Experimental Results and Discussion

### 3.1. Experimental and Simulation Results of Phase Change Asphalt Mixtures in Terms of Indoor Thermoregulation Performance

#### 3.1.1. Experimental Results and Analysis of Phase Change Asphalt Mixture Temperature Regulation Performance

**Figure 5:**
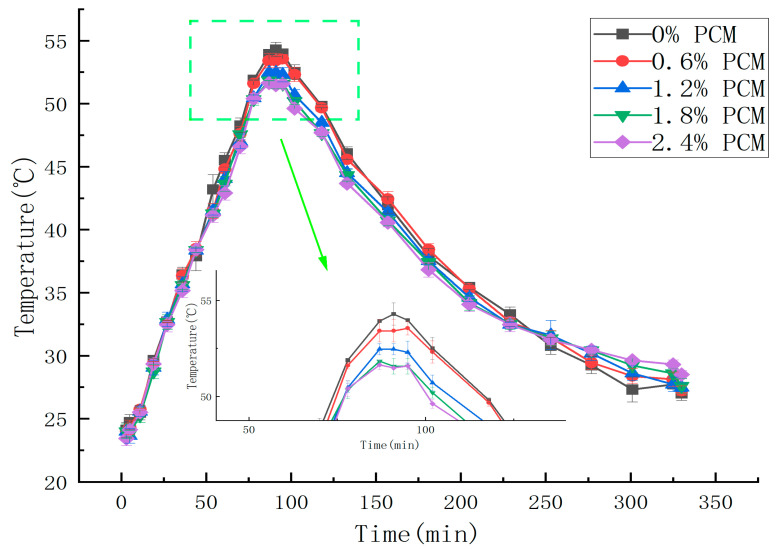
Experimental results of time–temperature variation with time for different PCM contents.

In order to simulate the actual situation of asphalt pavement at high temperatures in the summer, this study put the test specimens of each mixture together in an oven at 23 °C so that the temperature was stable. Then, the oven warmed the mixture specimens from 23 °C to 60 °C, simulating the process of asphalt pavement warming. When the sample temperature stabilized, the warming was stopped; then, the specimens were removed from the oven and left to cool naturally in the room at 23 °C to simulate the cooling process of the road surface. The temperature change in the mix specimen during this process was recorded using a temperature recorder; a temperature rise and fall process of 330 min was a temperature cycle, and the temperature collection time interval was 1min. Figure 5 shows the data obtained from a temperature cycle test. As can be seen in the figure, the peak internal temperature of the specimen is about 86min, at which time the specimen without phase change material reached the maximum temperature of 52.9 °C, the maximum temperature of the specimen with 0.6% of PCM content is 52.7 °C, the temperature of the specimen with 1.2% of PCM content is 51.6 °C, and the maximum temperature of the asphalt pavement with PCM content of 1.8% and 2.4% are 50.8 °C and 50.5 °C, respectively. Therefore, PCM doping can reduce the temperature peaks. As the mass ratio of phase change material increases, the temperature peak decreases. The temperature increase rate is shown in Table 9. The temperature increase rate of the specimens with 0.6% phase change material doping is not much different from that of the specimens without phase change material doping, whereas in specimens doped with 1.2% and above the mass ratio, the temperature increase rate decreases with the increase in mass ratio. This indicates that the doping of phase change materials plays a role in not only weakening the peak temperature but also reducing the rate of temperature increase. 

It can be seen that when the content of the phase change material is low, such as when the content is less than 1.8%, the temperature regulation effect is not obvious. When the phase change material doping reaches a certain level, the peak of high temperature decreases, and with the increase in content, the peak decreases more. This indicates that the phase change material plays a role in the asphalt and absorbs heat. As the content increases, the rate of temperature increase slows down, indicating that the phase change material also slows the heating and weakens the temperature stress. However, the actual test can be inaccurate because of various factors, such as the position of the specimen in the oven and the uniformity of the phase change material within the mix specimen. It was shown in [27] that when polyethylene glycol polyacrylamide graft copolymer phase change material (PPGC-PCM) was added at 7.5%, the maximum temperature of the pavement in summer was reduced by 8.9 °C compared with ordinary asphalt pavement, and the warming time was extended by 60 min.

In this experiment, the heating rates of different specimens are different before the temperature reaches the working temperature of the PCM. Thus, further temperature field simulations are needed to better investigate the thermoregulation effect of the PCM. In addition, in this experiment, the higher content of PEG1500 means there is higher content of calcium alginate as the carrier of the phase change material. Also, calcium alginate may affect the warming of the specimen, thus causing the temperature change near the probe to become faster with the increase in the content of the phase change composite. Thus, this study needs to consider calcium alginate as an influencing factor. However, according to Figure 5, the temperature changes were approximately the same when the temperature did not reach the PEG operating temperature. Therefore, it can be shown that the effect of calcium alginate on the temperature of the specimen is not significant when used as a carrier for the PCM, and thus the effect of calcium alginate in the subsequent test can be ignored. However, when applying the composite phase change material to asphalt pavement, it is necessary to consider its strength and other properties to ensure that the durability of the pavement is not damaged due to the incorporation of the phase change material.

#### 3.1.2. Simulation Results and Analysis of Phase Change Asphalt Mixture Temperature Regulation Performance

**Figure 6:**
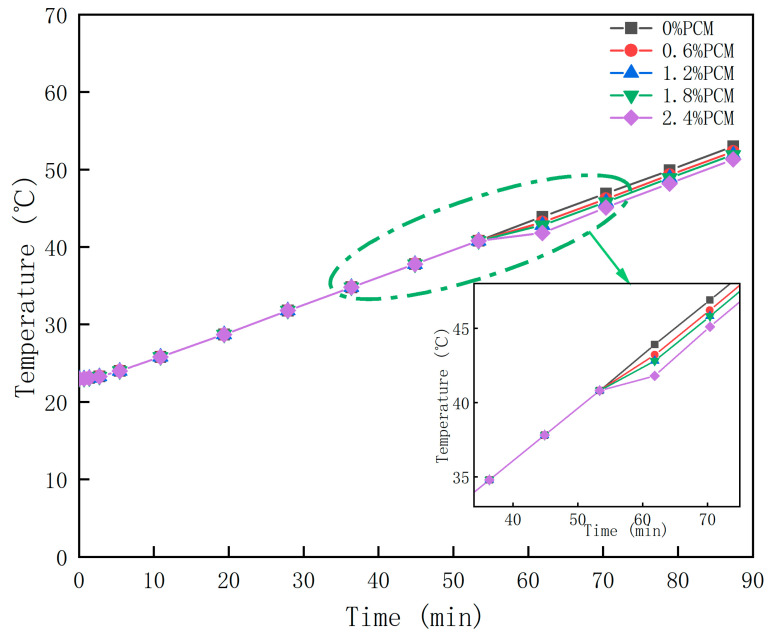
Simulation results of time–temperature over time for different PCM doping levels.

According to the data of the phase change temperature interval and latent heat of the phase change and other data for transient calculations, the probe temperature was obtained, as shown in Figure 6. From Figure 6, it can be seen that the warming trend of specimens containing different proportions of PCM is basically the same. Before the specimens reached 41 °C, i.e., the period ranging from the beginning of heating to 45 min, all groups had the same warming rate. However, in the 2.4% PCM group, it can be clearly seen that the warming rate starts to decrease slowly from 41 °C onwards. In the 0.6% and 1.2% specimens, the decrease in the warming rate was not significant. In the test group containing 1.8% PCM, there was a slight decrease in the warming rate. At a specimen temperature of 43 °C, the warming rate slowly returned to its original level until the end of the 85-min warming period. At the end of the warming period, the final temperatures of the different groups were not very different, but they were different. The specimens without phase change material had a final temperature of 52.0 °C, while the specimens with 2.4% PCM had a final temperature of 50.2 °C. The final temperature of the specimens with 2.4% PCM was 50.2 °C.

The results of the actual test (Figure 5) and those of the simulation test (Figure 6) are more consistent, and the pattern of the probe temperature change with the ambient temperature is basically the same for both tests. The PCM does play a role in absorbing heat and then slowing the warming, and this effect becomes more and more obvious as the doping amount increases. However, in the final temperature, the cooling effect is not obvious, and there is only a 1.8 °C difference between the specimen without phase change material and that with 2.4% phase change material. Compared with other temperature reduction measures, using PEG1500 as the phase change material in SMA-13 asphalt mixture specimens was not effective for indoor high-temperature conditioning. It is noteworthy that in the 2.4% PCM group, the warming rate returned to its original level at 42 °C, which indicates that the PCM stopped cooling at that point. However, the phase transition temperature interval of PEG1500 is 41–46 °C. Theoretically, the heating rate should return to the level before the phase transition at 46 °C. Therefore, it can be inferred that all PCMs in the specimen completed the phase change heat absorption process when the temperature reaches 42 °C. The reason for this could be the low content of phase change material. Therefore, with the goal of not affecting the road performance, the doping amount of the composite phase change material can be increased to realize a better cooling effect.

#### 3.1.3. Comparative Analysis of Experimental Results and Simulation Results of Phase Change Asphalt Mixture Temperature Regulation Performance

**Figure 7:**
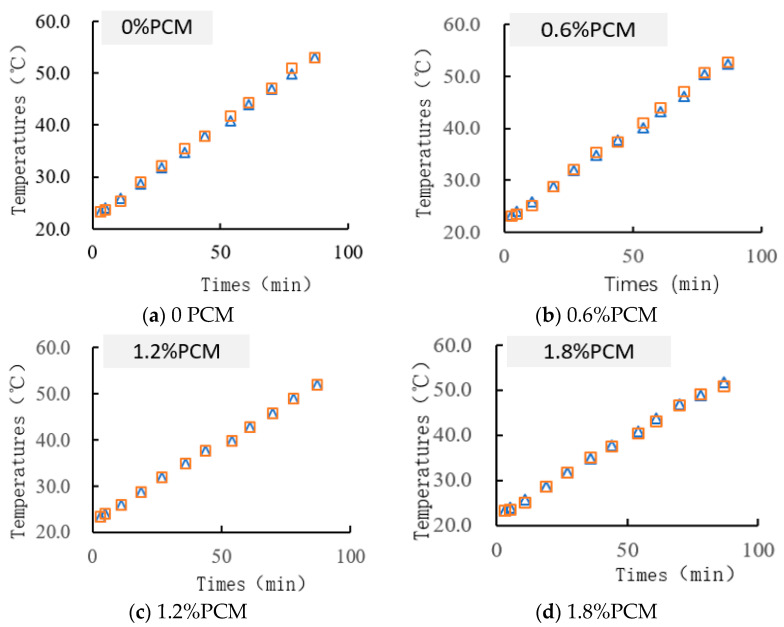

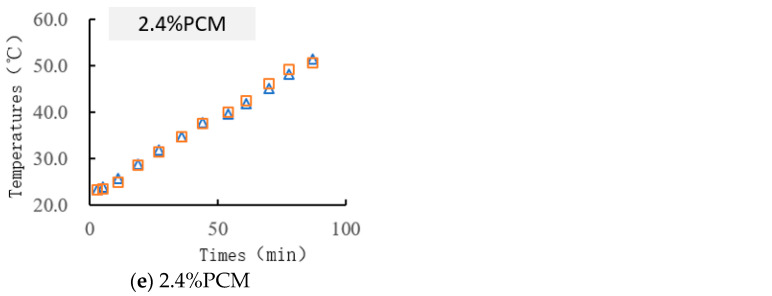
Comparison of experimental and simulated temperatures.

Figure 7 shows the 87 min test results and control table of Comsol’s probe temperature numerical calculation of different time temperatures. Figure 7a shows no phase change material added, Figure 7b shows 0.6% phase change material added, Figure 7c shows 1.2% phase change material added, Figure 7d shows 1.8% phase change material added, and Figure 7e shows 2.4% phase change material added. It can be seen that the finite element calculation results and the actual test results of the temperature differ by a maximum value of 1.4 °C, and a temperature difference of 1 °C or less accounted for 93.3% of the total number of finite element calculations for accurate results. Comparing the test and simulation results can also prove that the subsequent simulation results for the actual working conditions have a high degree of confidence.

### 3.2. Simulation Results and Analysis of Outdoor Temperature Field of Phase Change Asphalt Pavement

**Figure 8:**
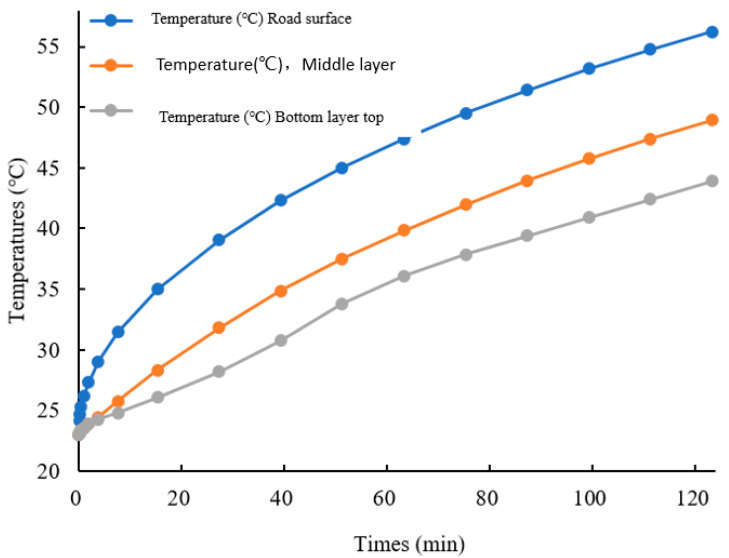
Temperature of the top surface of each layer of the 0PCM with time.

The Comsol Multiphisics 6.0 software heat transfer module was used to establish the transient study finite element model. Then, the surface layers without phase change material and with different rates of phase change material doping were compared and analyzed. The temperature of the top surface of each layer was the main object of concern because heat is transmitted vertically downward on the different layers, and the top surface is the first to receive the heat and has the highest temperature, which can better mimic the most unfavorable location of each layer. The simulation results are shown in Figure 8.

When no phase change material is added, the road surface temperature can reach up to 56 °C. At this point, the dynamic stability of the SBS asphalt starts to plummet and aggravates the aging, which leads to a decrease in the durability of the asphalt pavement. Not only does the top layer lose durability, but the middle layer is also within the range of the asphalt’s sudden drop in dynamic stability. Therefore, it is necessary to add phase change materials to temper the upper and middle surface layers, while the third layer has a lower temperature and was not considered for tempering in this study.

**Figure 9:**
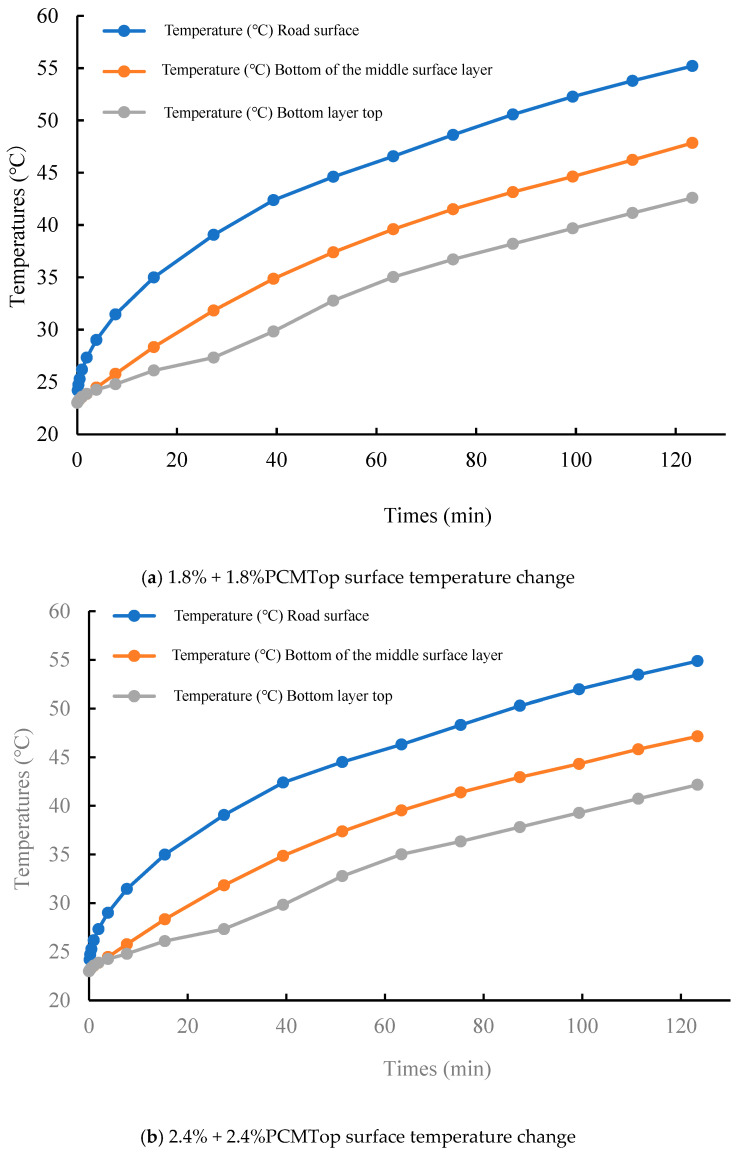
Variation in top surface temperature of asphalt pavement with different PCM dosages.

According to the results of the indoor high-temperature test, specimens with PCM content of 1.8% or less do not have a significant thermoregulation effect. Therefore, in the outdoor simulation, this study chose to add 1.8% or 2.4% PCM content in different layers to explore the appropriate dosage for the optimal thermoregulation effect. The surface temperatures of asphalt pavements after adding 1.8% and 2.4% phase change material to the upper and middle layers are shown in Figure 9.

Figure 9a shows that after adding 1.8% PCM to the top layer, the maximum temperature is 55.2 °C at 123 min, which is about 1 °C lower compared with the specimen without phase change material. However, it is still in the range of the sudden drop in dynamic stability, so the temperature regulation effect of asphalt pavement with 1.8% PCM doping is not ideal. Therefore, the phase change material content of the top layer was increased to 2.4%, and the simulated values are shown in Figure 9b. At 123 min, the maximum temperature of 54.8 °C is outside the range of a sudden drop in dynamic stability, so the PCM at this doping level has a certain thermoregulatory effect on phase change asphalt pavement.

The final temperature of the middle surface layer was about 1 °C lower than that without the phase change material after the phase change material was added, and 1.8 °C lower at 2.4% doping. It can be seen that the phase change material plays a role in temperature regulation, reduces the maximum temperature of the pavement, and slows the warming rate, and the temperature regulation effect is better when the dosage is 2.4%.

## 4. Conclusions and Outlook

In considering asphalt mixture as a temperature-sensitive material, its performance, its service life, and pavement disease are closely related to temperature conditions. Through material selection and mixture design, this study conducted indoor testing and numerical analysis to assess the composite phase change material’s cooling mechanism and the cooling characteristics of the asphalt mixture. The following main conclusions were drawn:

(1) Based on the asphalt pavement materials, use requirements, and the phase change characteristics of the phase change materials, PEG1500 and calcium alginate were chosen as the core material and wall material, respectively, to prepare microencapsulated composite phase change materials for use in asphalt mixtures. The results show that the highest temperature of the specimen with 0.6% PCM content is 52.7 °C, and that of the specimen with 1.2% PCM content is 51.6 °C. Meanwhile, the maximum temperatures of asphalt pavements with 1.8% and 2.4% contents were 50.8 °C and 50.5 °C, respectively, and the increase in PCM dosing helped reduce the temperature peaks. In addition, PCM blending can also reduce the heating rate of the material, which is beneficial to asphalt mixtures in the summer, a high-temperature season—the higher the PCM content, the better the cooling effect.

(2) The composite phase change material is applied in asphalt pavement as a thermoregulation material. When the doping amount is 2.4%, its cooling effect can reach 1.5 °C, and at the same time, it can effectively reduce the warming rate of asphalt pavement.

(3) An indoor Marshall specimen warming test model was established in Comsol, and the test and simulation results are found to be similar. Thus, it is feasible to simulate the subsequent asphalt pavement test in Comsol, which can effectively save resources and costs.

(4) This study established an outdoor asphalt pavement temperature field simulation in Comsol. Without the addition of phase change materials, the road surface temperature reaches 56 °C, and the SBS-modified asphalt temperature maintains dynamic stability within the sudden-drop range, exacerbating the aging of the asphalt pavement. After adding 1.8% phase change material to the upper and middle surface layers, the temperature remains in the range of the dynamic stability drop; after adding 2.4% composite phase change material to the upper and middle surface layers, the pavement temperature is reduced to 54 °C and the warming rate is slowed.

Overall, the incorporation of phase change materials can be effective, but the cooling effect of PCM is not ideal at high temperatures for reasons that may be related to the errors generated using phase change materials, the selection of wall materials, and the effects of temperature in the experiments. Therefore, in the future, it is recommended to consider interdisciplinary cooperation among aerospace, chemistry, and architecture to expand the promotion of the application range of PCMs, with the aim of obtaining PCMs with better thermoregulation effects. Furthermore, this study verified the mechanism under ideal laboratory conditions. Future research will focus on building full-scale test sections to monitor the road surface response in different seasons and meteorological conditions (such as wind speed and rainfall) over the long term in order to quantify the impact of these environmental factors on performance and to establish more accurate prediction models.

## Figures and Tables

**Table 1:** Mineral and synthetic grading scale.

Mineral Specification	Proportions	Passage Rate (%) for the Following Mesh Sizes (mm)									
		16	13.2	9.5	4.75	2.36	1.18	0.6	0.3	0.15	0.075
10–15	40.0	100	78.85	8.59	0.05	0.05	0.05	0.05	0.05	0.05	0.05
5–10	34.0	100	99.88	90.25	6.71	0.72	0.52	0.52	0.52	0.52	0.52
0–3	16.0	100	100	100	95.22	69.47	51.38	33.44	21.71	13.3	3.97
Mineral powderpowder	10.0	100.0	100.0	100.0	100.0	100.0	100.0	100.0	97.6	91.4	83.9
Upper limit of gradation	100	100	100	75	34	26	24	20	16	15	12
Lower limit of gradation	100	100	90	50	20	15	14	12	10	9	8
Median of gradation	100	100	95	63	27	21	19	16	13	12	10
Synthetic gradation grade	100.0	100.0	91.5	60.1	27.5	21.3	18.4	15.6	13.5	11.4	9.2

**Table 2:** Basic properties of SBS-modified asphalt.

Item	Flat	Technical Requirements	Test Results	Test Methods
penetration of a needle (25 °C, 100 g, 5 s)	0.1 mm	60~800	67	T0604
ductility (5 cm/min, 5 °C)	cm	≥30	44	T0605
Softening point (global method)	°C	≥55	72.4	T0606
Densities (15 °C)	g/cm^3^	--	1.044	T0603
Elastic recovery 25 °C	%	≥75	98.0	T0662
mass loss	%	0.8	0.56	T0610
Residual needle penetration ratio (25 °C)	%	≥60	71.3	T0604
Residual elongation (10 °C)	cm	≥15	25	T0605

**Table 3:** SMA-13 Marshall test results.

Oil-Rock Ratio (%)	Theoretical Maximum Relative Density	Gross Volume Relative Density	VV (%)	VMA (%)	VFA (%)	VCAmix (%)	Degree of Stability (kN)	Stream Value (0.1 mm)
6.0	2.540	2.448	3.6	18.4	80.3	40.7	7.64	33

**Table 4:** SMA-13 Marshall test on proportion design—technical index table.

Enterprise	Work Unit	Test Results	Regulatory Requirement
void ratioVV	%	3.6	3~4
VCAmix	%	40.7	≤VCADRC
VMA	%	18.4	≥17.0
VFA	%	80.3	75~85
degree of stability	kN	7.64	≥6.0
stream value	0.1 mm	33	-

**Table 5:** SMA-13 mix target mix ratio test results.

Enterprise	Work Unit	SMA-13	Regulatory Requirement	Test Methods
Loss of binding material in asphalt segregation tests	%	0.06	≤0.1	T0732
Loss of mix for flyaway test (20 °C)	%	5.6	≤15	T0733
DS	times/mm	5863	≥3000	T0719
Cracking resistance at low temperature	µε	2844	≥2800	T0728
Residual Marshall Stability	%	91.2	≥80	T0709
Freeze-thaw split residual strength ratio	%	83.1	≥80	T0729
seepage coefficient	mL/min	-	≤80	T0730
tectonic depth	mm	0.96	0.8–1.5	T0731

**Table 6:** Basic material properties.

Densities (kg/m^3^)	Thermal Conductivity (W/m·K)	Constant Pressure Heat Capacity (J/K)
2540	2.3	1000

**Table 7:** Latent heat of phase transition for different PCMs.

Quantity Contained	0.6%	1.2%	1.8%	2.4%
latent heat of phase transition (KJ/kg)	14	28	42	56

**Table 8:** Asphalt pavement material parameters.

Matter	Thicknesses (cm)	Densities (kg/m^3^)	Thermal Conductivity (W/m·K)	Constant Pressure Heat Capacity (J/K)
SMA-13 upper layer	4	2128	2.3	1000
AC-20 middle layer	6	2540	1.55	1000
AC-25 lower layer	8	2580	1.6	1000

**Table 9:** Rising temperature rate.

	0 PCM	0.6% PCM	1.2% PCM	1.8% PCM	2.4% PCM
heating rate (°C/min)	0.60	0.60	0.59	0.58	0.57

## Data Availability

The original contributions presented in the study are included in the article; further inquiries can be directed to the corresponding author.
